# Transcutaneous spinal direct current stimulation increases corticospinal transmission and enhances voluntary motor output in humans

**DOI:** 10.14814/phy2.14531

**Published:** 2020-08-18

**Authors:** Tomofumi Yamaguchi, Mikkel M. Beck, Eva R. Therkildsen, Christian Svane, Christian Forman, Jakob Lorentzen, Bernard A. Conway, Jesper Lundbye‐Jensen, Svend S. Geertsen, Jens B. Nielsen

**Affiliations:** ^1^ Department of Neuroscience University of Copenhagen Copenhagen N Denmark; ^2^ Department of Physical Therapy, Faculty of Health Science Juntendo University Tokyo Japan; ^3^ JSPS Postdoctoral Fellow for Research Abroad Tokyo Japan; ^4^ Department of Nutrition, Exercise and Sports (NEXS) University of Copenhagen Copenhagen N Denmark; ^5^ Elsass Foundation Charlottenlund Denmark; ^6^ Department of Biomedical Engineering University of Strathclyde Glasgow UK

**Keywords:** movement, spinal stimulation, transcranial magnetic stimulation

## Abstract

Optimization of motor performance is of importance in daily life, in relation to recovery following injury as well as for elite sports performance. The present study investigated whether transcutaneous spinal direct current stimulation (tsDCS) may enhance voluntary ballistic activation of ankle muscles and descending activation of spinal motor neurons in able‐bodied adults. Forty‐one adults (21 men; 24.0 ± 3.2 years) participated in the study. The effect of tsDCS on ballistic motor performance and plantar flexor muscle activation was assessed in a double‐blinded sham‐controlled cross‐over experiment. In separate experiments, the underlying changes in excitability of corticospinal and spinal pathways were probed by evaluating soleus (SOL) motor evoked potentials (MEPs) following single‐pulse transcranial magnetic stimulation (TMS) over the primary motor cortex, SOL H‐reflexes elicited by tibial nerve stimulation and TMS‐conditioning of SOL H‐reflexes. Measures were obtained before and after cathodal tsDCS over the thoracic spine (T11‐T12) for 10 min at 2.5 mA. We found that cathodal tsDCS transiently facilitated peak acceleration in the ballistic motor task compared to sham tsDCS. Following tsDCS, SOL MEPs were increased without changes in H‐reflex amplitudes. The short‐latency facilitation of the H‐reflex by subthreshold TMS, which is assumed to be mediated by the fast conducting monosynaptic corticomotoneuronal pathway, was also enhanced by tsDCS. We argue that tsDCS briefly facilitates voluntary motor output by increasing descending drive from corticospinal neurones to spinal plantar flexor motor neurons. tsDCS can thus transiently promote within‐session CNS function and voluntary motor output and holds potential as a technique in the rehabilitation of motor function following central nervous lesions.

## Graphical abstract

We aimed to investigate the effects of transcutaneous spinal direct current stimulation (tsDCS) on descending activation of spinal motor neurons in healthy adults. Cathodal tsDCS facilitated peak torque and acceleration of a ballistic motor task and increased both the motor evoked potentials of the soleus, and the short‐latency facilitation of the soleus H‐reflex. Therefore, we argue that tsDCS may be a useful technique for facilitating descending drive and promoting motor function following central nervous lesions.

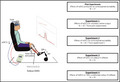

## 1 INTRODUCTION

The spinal cord contains a complex network of neural circuitries, which is capable of generating basic patterns of motor activity in the absence of descending control from supraspinal centers (Kiehn, 2006). In humans, such activity is rarely seen following complete spinal cord lesions and attempts to facilitate spinal network activity below the lesion through pharmacological agents (Domingo, Al‐Yahya, Asiri, Eng, & Lam, 2012), training (Harkema et al., 2012; Hubli & Dietz, 2013) or epidural electrical spinal cord stimulation (Roy, Harkema, & Edgerton, 2012) have not been sufficiently successful in this patient group to make their way into routine clinical use. However, there is accumulating evidence to suggest that it is possible to re‐gain considerable function through training if a minimum of descending connections have survived and if the neural circuitries below the lesion are modulated either pharmacologically or through electrical stimulation during functional training (Gill et al., 2018; Roy et al., 2012; Taccola, Sayenko, Gad, Gerasimenko, & Edgerton, 2018). This indicates that the effect of the spinal stimulation depends crucially on survival of some descending connectivity from supraspinal centres and probably relates to concurrent plastic changes in the interaction of cortical and spinal circuitries.

In these studies, electrical stimulation has been applied directly to the spinal cord through epidural electrodes, which has the advantage of proximity to the spinal circuitries, but also involves relatively extensive surgery and may not always be well tolerated by the patient (Gill et al., 2018; Taccola et al., 2018). For clinical purposes, it would be desirable if the stimulation could be applied non‐invasively and at intensities that are not painful. Lately, considerable interest has therefore been devoted to the possibility of modulating spinal neural transmission by transcutaneous application of a constant electrical direct current (DC) over the spinal cord; tsDCS (Berry, Tate, & Conway, 2017; Jankowska, 2017; Murray, Tahayori, & Knikou, 2018; Song & Martin, 2017). This has been spurred on by the demonstration that cortical DC stimulation may modulate cortical excitability and induce lasting plastic changes in cortical circuitry raising prospects of a clinical role in neurorehabilitation following brain lesion (Lefaucheur et al., 2017; Nitsche et al., 2008; Nitsche & Paulus, 2000). Importantly, tsDCS over lumbar spinal segments has recently been shown to facilitate voluntary activation of leg muscles and improve motor performance in a functional motor task meriting a role for its clinical application (Berry et al., 2017).

DC stimulation of the spinal cord has a long history. It was already shown in the early 1960s by John Eccles and colleagues that low level DC stimulation could alter the excitability of axons in the cat spinal cord in a polarity‐dependent, bidirectional manner (Eccles, Kostyuk, & Schmidt, 1962). With electrodes placed ventrally and dorsally in direct contact with the spinal cord, dorsal axons (Ia afferents) were demonstrated to be depolarized under a cathode and hyperpolarized under an anode. These findings have now been confirmed in rat experiments in which direct recordings have been made from ascending fibers in the dorsal columns of the spinal cord (Baczyk & Jankowska, 2018; Jankowska, Kaczmarek, Bolzoni, & Hammar, 2017). Importantly, the excitability changes outlast the stimulation and may be seen for several min to hours following relatively brief application of the electrical field (Baczyk & Jankowska, 2018; Kaczmarek, Ristikankare, & Jankowska, 2017).

The interpretation of transcutaneous spinal DC stimulation in humans is more complex than in animals since the electrodes are placed at a considerable distance from the spinal cord and the applied current has to penetrate skin, subcutaneous tissue, muscle and bones before reaching the spinal cord. This probably explains why higher intensities of stimulation are necessary in human subjects and that a considerable variability has been found between subjects and between studies. Despite this, motor evoked potentials (MEPs) elicited by transcranial magnetic stimulation (TMS) of the primary motor cortex have been shown consistently to be facilitated by cathodal tsDCS and diminished by anodal tsDCS over the spinal segments where the motor neurons of the investigated muscle are located (Bocci, Barloscio, et al., 2015; Bocci, Marceglia, et al., 2015; Knikou, Dixon, Santora, & Ibrahim, 2015). Significantly, in the majority of studies a failure to demonstrate any changes in H‐reflexes, which, at least in part, probes the excitability of the spinal motor neurons has been reported (Albuquerque, Mendonca, Campelo, Shirahige, & Monte‐Silva, 2018). This suggests somewhat surprisingly that the functional effect of tsDCS is not necessarily related to effects on spinal motor neurons and interneurons locally, but may rather be related to a facilitation of corticospinal drive through increased excitability of corticospinal neurons. The observation that short‐latency cortical inhibition (SICI) measured in a paired‐pulse TMS paradigm is also affected by tsDCS over the lumbar spinal cord is consistent with the hypothesis of a role of the primary motor cortex (M1) (Bocci, Barloscio, et al., 2015). However, more studies are needed to delineate the specific mechanisms by which tsDCS exerts its effects.

The purpose of the present study was to investigate whether tsDCS over the L1‐L2 spinal segments enhances voluntary muscle activation through increased corticospinal drive to spinal motor neurons located more caudally. We report that cathodal tsDCS increases voluntary EMG and acceleration during ballistic plantarflexion contractions. This is accompanied by increased MEPs and short‐latency facilitation of the soleus H‐reflex induced by TMS, without any changes in the H‐reflex. This suggests that tsDCS delivered at rostral lumbar levels enhances corticospinal drive to spinal motor neurons at more caudal levels and thereby promotes functional ability.

## 2 METHODS

### 2.1 Ethical approval and enrolled participants

Forty‐one neurologically intact, able‐bodied human adults (20 male; 24.0 ± 3.2 years) participated in the study. The study was approved by the ethics committee of the capital region of Copenhagen, Denmark (H‐17019671). Participants provided written consent prior to participation upon receiving thorough oral and written information. The experimental procedures conformed to the Declaration of Helsinki.

### 2.2 Experimental design

**Figure 1:**
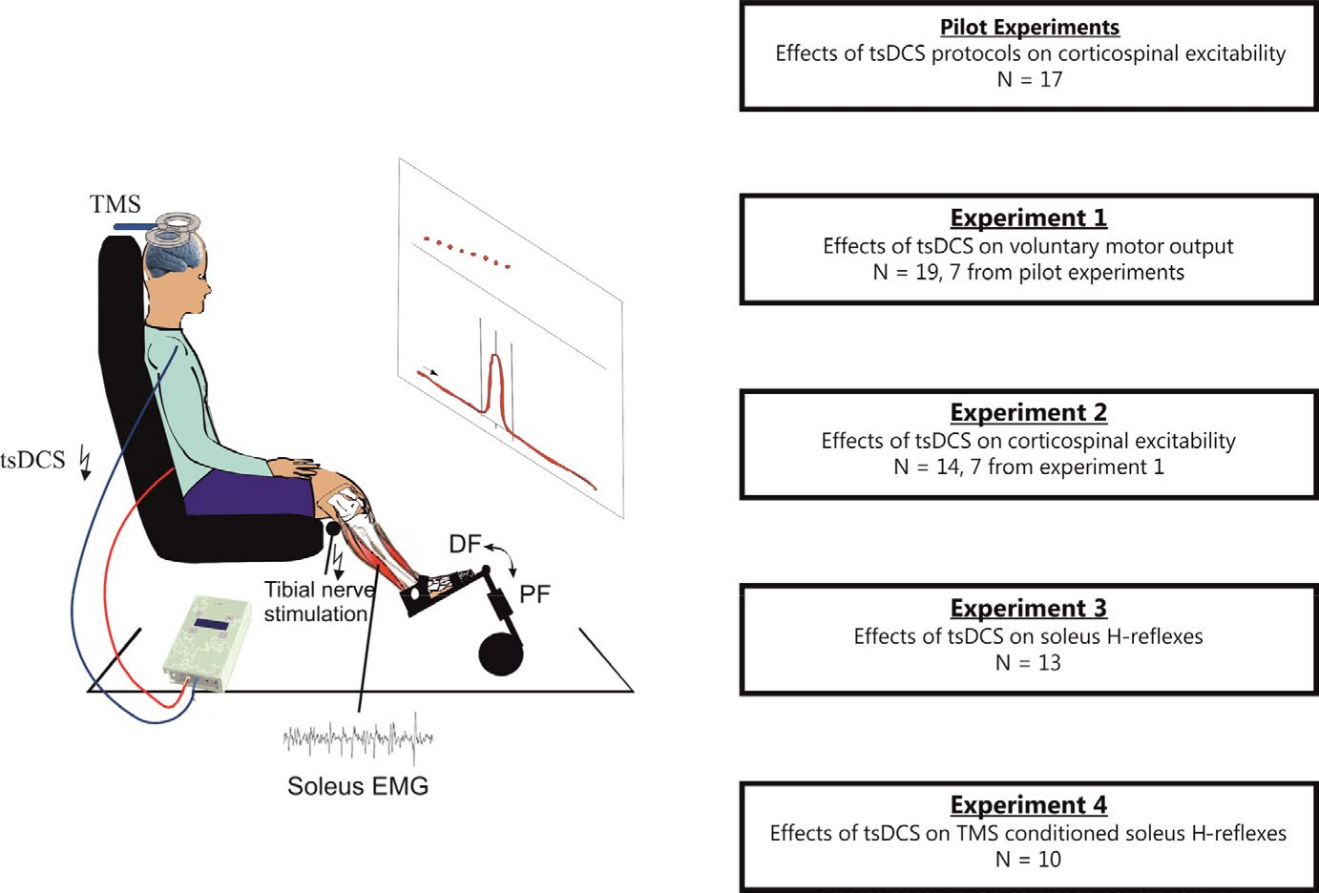
Experimental design and setup

The study consisted of two pilot experiments to determine the most appropriate tsDCS stimulation protocol and four experiments designed to evaluate the acute effects of tsDCS on voluntary muscle activation and the potential mechanisms of action (Figure 1). The pilot experiments are described in the Supplementary Material. These experiments were performed in 17 participants and systematically investigated the effect of different tsDCS stimulation intensities and durations on the amplitude of Soleus MEPs. It was determined that tsDCS with the cathode over the lumbar segments, a stimulation intensity of 2.5 mA and a stimulation duration of 10 min produced clear and reproducible effects (Figure S1) and these stimulation parameters were therefore used in subsequent experiments. In experiment 1, we investigated the effects of tsDCS on voluntary motor output using a ballistic task involving maximal activation of the ankle plantar flexor muscles. In experiment 2, the effects of tsDCS on corticospinal excitability were evaluated by TMS over the primary motor cortex (M1). In experiment 3, we investigated the effects of tsDCS on unconditioned soleus H‐reflex amplitudes. Lastly, in experiment 4, we explored potential pathway‐specific plasticity of tsDCS by conditioning soleus H‐reflexes with subthreshold TMS. For all experiments, participants were comfortably seated in a custom‐build chair with their right foot placed on a footplate and secured with adjustable straps (Figure 1). The participant sat with the hip semi‐flexed (70–80 degrees), the knee flexed (70–80 degrees) and the ankle dorsi‐flexed (10 degrees) and this position was maintained throughout all the experiments.

### 2.3 Transcutaneous spinal direct current stimulation (tsDCS)

tsDCS was delivered using a NeuroConn DC Stimulator Plus (Neurocare group GMBH, Germany) through two 5 × 7 cm carbon rubber electrodes with the cathode mounted at the T11‐T12 spine segments and the anode positioned on the lateral side of the right shoulder. Electrodes were covered with electroconductive gel (Signagel, Parker Laboratories) to improve impedance levels (kept below 10 kΩ during stimulation) and fastened using surgical tape. Stimulation intensity and duration varied across experiments in relation to the specific hypothesis of the respective experiment.

### 2.4 EMG recordings

EMG was recorded from the soleus muscle using bipolar electrodes (BlueSensor, Ambu, Ølstykke, DK) placed over the muscle belly with an inter‐electrode distance of approximately 2 cm. EMG signals were amplified ×5000, bandpass filtered 5‐1000 Hz (Digitimer Ltd) and digitized at 2 kHz (CED1401+, Signal v. 7.02 software, Cambridge Electronic Designs, Cambridge, UK). Data were stored on a PC for offline analyses.

### 2.5 Transcranial magnetic stimulation (TMS)

TMS was applied over the M1 using a Magstim 200 stimulator (Magstim Company Ltd.) connected to a figure‐of‐eight coil with a loop‐diameter of 7 cm. At the beginning of each experiment, the coil was systematically moved to the spot that consistently evoked the largest MEPs in the soleus muscle of the respective participant at rest (i.e., the motor hotspot), and the position of the coil was registered using Brainsight® frameless stereotactic neuronavigation system (Brainsight‐Frameless 2.3 Rogue Research, Canada). Next, the resting motor threshold (rMT) was found by altering the stimulation intensity on the magnetic stimulator until an intensity that elicited MEPs with an amplitude of at least 50 µV in 5/10 cases for the soleus muscle was reached. A stimulus intensity of 1.2 rMT was then used throughout the experiment before and after tsDCS (see below).

### 2.6 Peripheral electrical nerve stimulation

Soleus H‐reflexes were evoked by electrical stimulation (1 ms pulses, DS7A, Digitimer Ltd.) of the tibial nerve using a ball‐shaped Simon electrode strapped to the popliteal fossa as the cathode, and saline‐soaked rectangular electrode placed just proximal of the patella as the anode.

### 2.7 Experiment 1: Effects of cathodal tsDCS on voluntary motor output

To investigate whether tsDCS changed voluntary motor activation, 19 healthy individuals were enrolled in a double‐blinded, sham‐controlled cross‐over study performed on two days separated by at least a week. Cathodal tsDCS was delivered at 2.5 mA for 10 min using the setup described above. Sham tsDCS was performed by applying a 30‐s stimulation ramp at 2.5 mA in the beginning and in the end of the stimulation duration. Voluntary motor output was assessed using a ballistic motor task involving the ankle muscles similar to Lundbye‐Jensen, Petersen, Rothwell, & Nielsen, 2011 (Lundbye‐Jensen et al., 2011). Participants were instructed to perform a maximal voluntary plantar flexion against a force pedal while seated with the highest possible acceleration in response to an auditory and visual GO‐cue and subsequently return to the initial resting position within a total time window of 500 ms. This task has previously been used to assess ballistic motor performance and learning (Lundbye‐Jensen et al., 2011). During these sessions, the participants received no visual feedback regarding their motor performance. Participants performed two blocks of 10 trials with each trial lasting 10 s prior to and three blocks following (2, 10 and 20 min) tsDCS or sham. At the outset of the experiment and immediately following tsDCS or sham, the participants performed three test contractions to become accustomed to the task. Voluntary motor output was quantified as the peak acceleration averaged over ten trials, and expressed relative to the first baseline measure. Additionally, peak rectified soleus EMG for the first 100 ms of EMG onset (defined as increases of 50 µV above baseline EMG) was determined as a measure of voluntary muscle activation to complement the behavioral outcomes.

### 2.8 Experiment 2: Effects of tsDCS on corticospinal excitability

Fourteen healthy individuals, from which four participated in experiment 1, were enrolled in a within‐subject designed experiment, in which the effects of tsDCS on the excitability and transmission in the corticospinal system were quantified by the amplitude of Soleus MEPs. Corticospinal excitability was examined by applying 15 single TMS pulses of 1.2× soleus rMT prior to (~ −5 and −2 min) and following tsDCS (2, 10, 20, and 30 min after). The average value of the peak‐to‐peak soleus MEP amplitudes at each time point following tsDCS was expressed as a percentage of the first baseline measure (−5 min) and used for statistical analysis.

### 2.9 Experiment 3: Effects of tsDCS on soleus test H‐reflexes

Next, we investigated whether cathodal tsDCS led to changes in the size of the soleus H‐reflexes. Thirteen healthy individuals underwent cathodal tsDCS at 2.5 mA for 3 min in order to investigate acute effects of the stimulation on the Soleus H‐reflex. H‐reflexes measured before (−5 min) and 2 min after tsDCS. In the beginning of each experiment, the size of the maximum soleus motor response (M_max_) was measured, and the subsequent electrical stimulations were delivered at a fixed intensity adjusted to approximately ~20% of the initial M_max_ to evoke H‐reflexes on the ascending part of the H‐reflex recruitment curve at baseline. This tsDCS protocol was adopted based on the results from the previous experiments, suggesting that tsDCS‐induced effects are most prominent just after the cessation of DC stimulation.

### 2.10 Experiment 4: TMS conditioning of the soleus H‐reflex

In 10 able‐bodied individuals, who also participated in experiment 1 and 2, pathway‐specific effects of cathodal tsDCS were investigated. This was done using subthreshold TMS‐conditioning of the soleus H‐reflex, permitting a separation of short‐latency monosynaptic facilitation from longer‐latency effects. The H‐reflex was evoked by brief (1 ms) electrical pulses, and the Soleus motor hotspot was located using the same setup as described above. The TMS stimulation intensity was subsequently reduced to 5% below RMT such that TMS produced EPSPs in the spinal motor neurons without eliciting a MEP. Next, time courses of the conditioning effects of TMS on the test H‐reflex were obtained for each individual by testing different interstimulus intervals from −5 to 0 ms (negative values indicating the delivery of a TMS single‐pulse after the electrical stimulation to evoke an H‐reflex) (See Figure 5a). The earliest ISI consistently displaying a conditioned facilitation was chosen to reflect short‐latency facilitation (SLF) and kept for the remainder of the experiment. Then, unconditioned test H‐reflexes and TMS‐conditioned H‐reflexes were evoked in a randomized order in blocks consisting of 15 stimulations, twice before (−10, −5 min) and three times after (2, 10 and 20 min) 10 min of 2.5 mA cathodal tsDCS. The size of the test H‐reflex was kept at ~20% of M_max_.

### 2.11 Statistical analysis

For statistical analyses the open‐access software RStudio was used (R Core Team, 2018). In accordance with the experimental designs, linear mixed effect models were fitted to the dependent variable of the studies with an interaction between condition and time (Condition X Time) unless otherwise stated. Using mixed effect models are comparable to two‐way RM ANOVAs, but allows flexible model fitting in cases where single data points are missing. Furthermore, it enables introduction of random effects that helps controlling for unobserved heterogeneity. As random effects, we entered intercepts for the individual participant. This was added to account for parts of the inherent inter‐subject variability to reduce residual errors of the model. Linear mixed effect models were fitted using the *lme4* package (Bates, Maechler, Bolker, & Walker, 2014). To ensure model validity, we visually inspected residual plots and normal probability plots to control for overt nonconformity from homoscedasticity or normality, respectively. Model‐based planned comparisons evaluating the specific hypotheses tested were computed using the *multcomp* package (Hothorn, Bretz, & Westfall, 2008). To account for statistical multiplicity, these pairwise comparisons were corrected using the single‐step method. A significance level of 0.05 was applied. Data in figures are presented as means ± *SD*, unless otherwise stated, and model estimates in the main text are presented with standard error of the estimate.

## 3 RESULTS

### 3.1 Experiment 1: Effects of tsDCS on voluntary motor output

**Figure 2:**
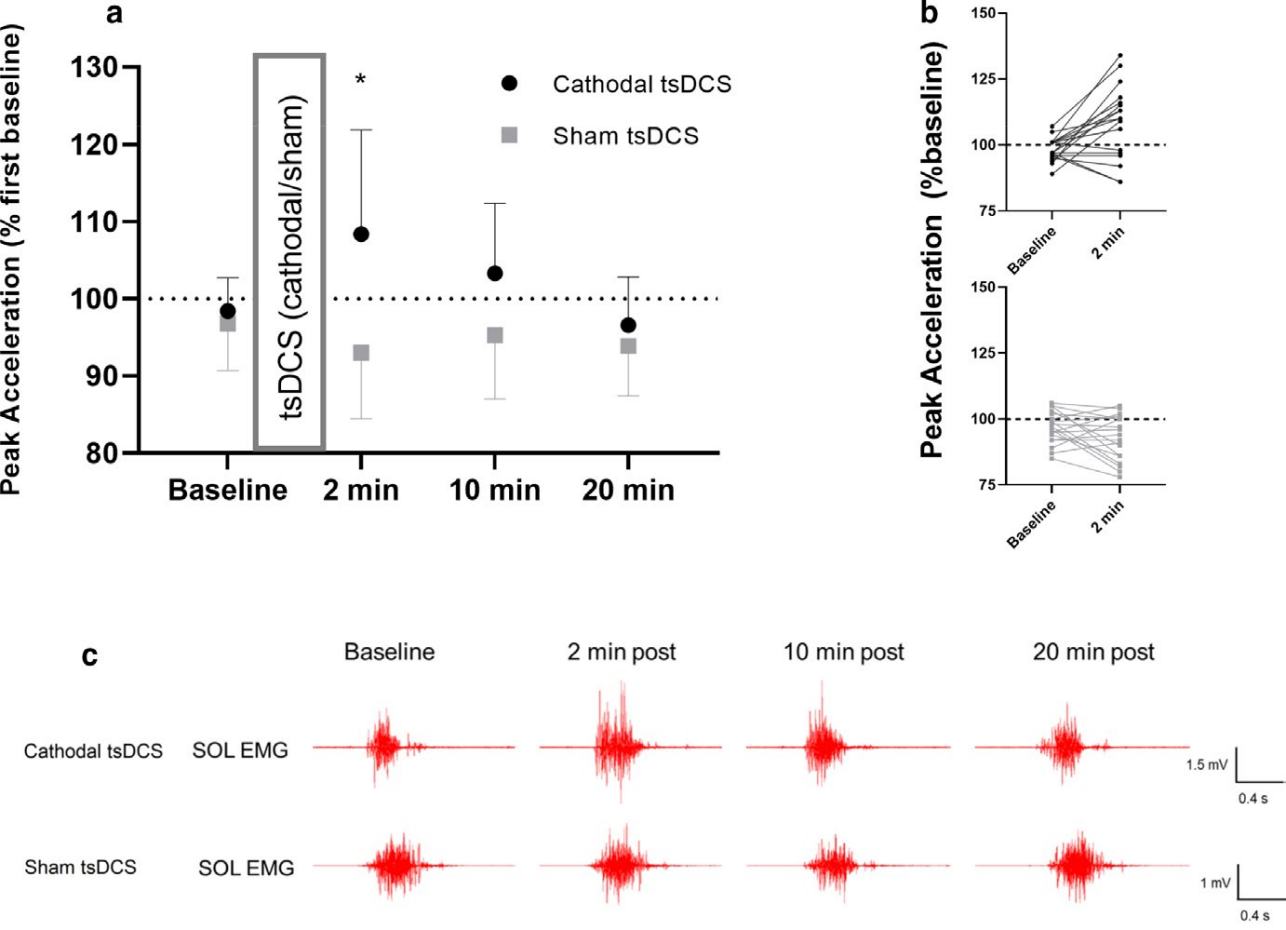
Effects of tsDCS on voluntary motor output. (a) Group data (*n* = 19) displaying peak acceleration during a ballistic plantar flexion before (baseline) and following (2, 10 and 20 min) cathodal tsDCS or sham. (b) Individual changes in peak acceleration during ballistic plantar flexion before (baseline) and immediately after (2 min) tsDCS. (c) Soleus EMG recorded from a single representative participant before (baseline) and after cathodal tsDCS or sham. Group results are expressed as means ± *SD*. * indicates significant between‐group differences in changes in voluntary motor output (*p* < .05)

First, we investigated whether tsDCS enhanced voluntary muscle activation of the ankle plantar flexors. We hypothesized that cathodal tsDCS would lead to a greater improvement in peak acceleration and maximal force production compared to sham stimulation, and that increased EMG, reflecting the voluntary output from the alpha motor neurons, would parallel this. The changes in peak acceleration were also greater following cathodal tsDCS compared to sham from baseline to 2 min post tsDCS (18.2 ± 3.7%, *p* < .001) (Figure 2a). Increases in peak acceleration were observed in 68% of participants after tsDCS, but only in 32% of participants following sham stimulation (Figure 2b). Furthermore, significant changes in rectified EMG were present at 2 min (*p* = .02) and marginally significant changes were observed at 10 min (*p* = .06) following tsDCS, but not following sham (*p*‐value range = 0.50–0.91) (Figure2c). These findings indicate that cathodal tsDCS increases maximal voluntary motor output and improves performance in a motor task requiring ballistic plantar flexions.

### 3.2 Experiment 2: Effects of tsDCS on corticospinal excitability

**Figure 3:**
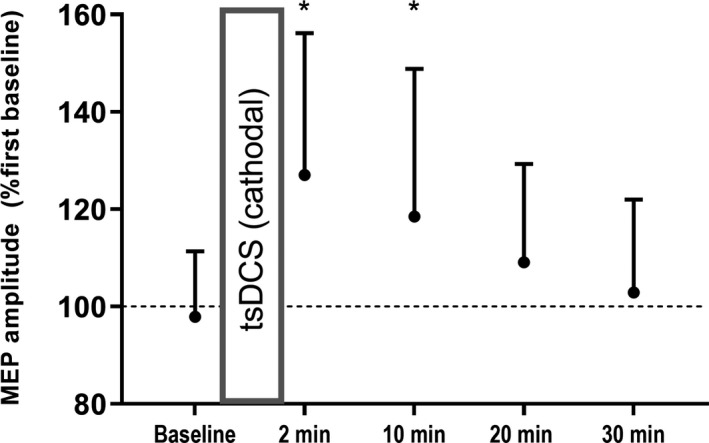
Effects of cathodal tsDCS on corticospinal excitability. Group data (*n* = 14) representingthe normalized MEP amplitude before and after 2.5 mA cathodal tsDCS for 10 min. Results are expressed as means ± *SD*. * indicates significant within‐condition differences in MEP amplitude from baseline (*p* < .05)

To investigate the mechanisms underlying the behavioral effects we explored whether tsDCS induced changes in soleus MEPs elicited by TMS over the leg area of the M1. As can be seen from Figure 3, tsDCS (2.5 mA for 10 min) increased the MEPs for 2 min (29.1 ± 7.1%, *p* < .001) and 10 min after tsDCS (20.6 ± 7.1%, *p* = .01).

### 3.3 Experiment 3: Effects of tsDCS on soleus H‐reflexes

**Figure 4:**
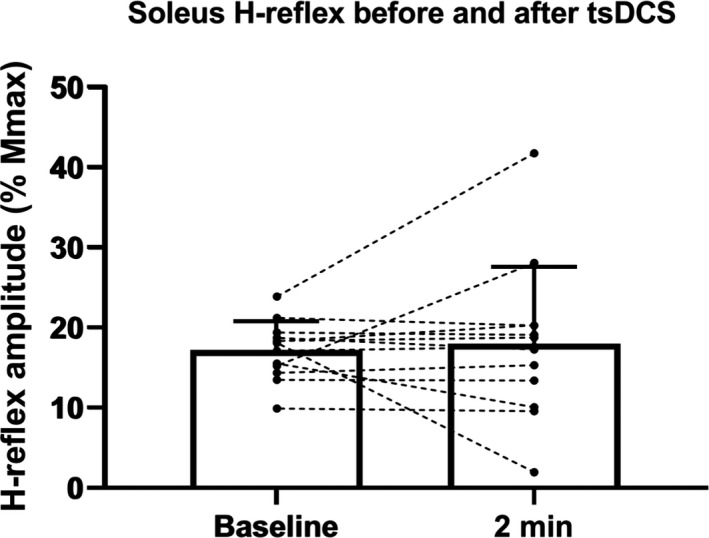
Effects of tsDCS on soleus H‐reflexes. Group data (*n* = 13) with individual paired traces for H‐reflex amplitudes before and two min after cathodal tsDCS

Another experiment was performed to investigate whether tsDCS led to changes in test H‐reflex size. We compared H‐reflex sizes before and after a session of tsDCS. H‐reflex amplitudes did not change following cathodal spinal DC stimulation (*p* = .35) (Figure 4). There were also no changes in M‐responses to the same stimulation (*p* = .65) or in Mmax elicited by supramaximal stimulation. This suggests that tsDCS potentiates networks upstream of the motor neuron pool, potentially involving an increased descending corticospinal drive to spinal motor neurons. This was tested in experiment 4.

### 3.4 Experiment 4: Effects of tsDCS on TMS conditioned soleus H‐reflexes

**Figure 5:**
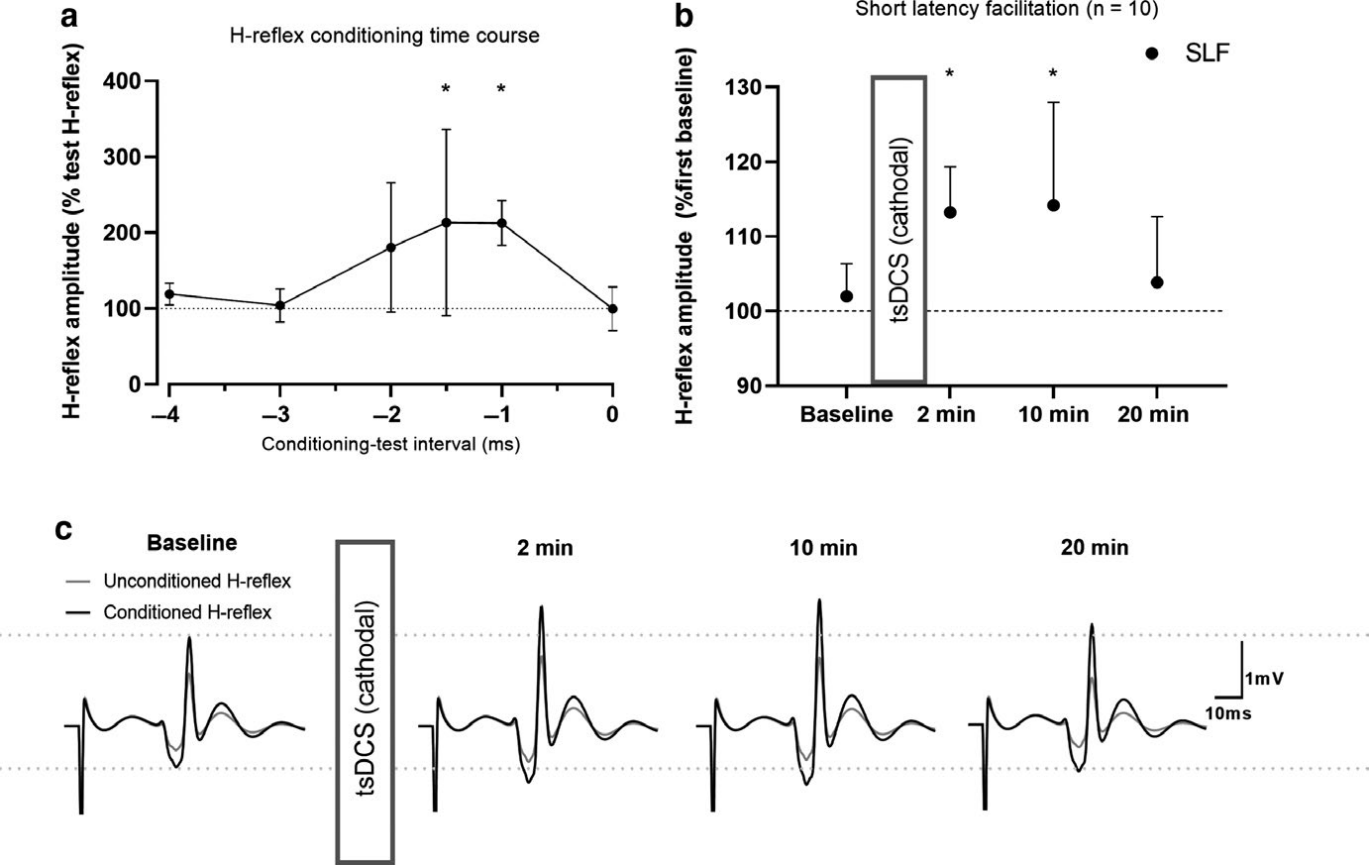
Effects of tsDCS on short‐latency facilitation of the soleus H‐reflex. (a) Represents the time course used to determine the conditioning‐test interval for short‐latency facilitation (SLF) of the H‐reflex of a single representative participant. An H‐reflex was evoked at 0 ms by brief (1 ms) electrical stimulation of the tibial nerve. A conditioning, subthreshold TMS pulse was delivered at different time points after H‐reflex elicitation. Negative conditioning‐test intervals correspond to the delivery of a TMS pulse after the electrical stimulation leading to an H‐reflex. The conditioned H‐reflex amplitude was logged for different conditioning‐test intervals and compared to the test (unconditioned) H‐reflex amplitude. The earliest conditioning‐test intervals with a significant facilitation was chosen for each participant (in a, 1.5 ms was chosen). *corresponds to a significant difference from test H‐reflex (*p* < .05). (b) Group data (*n* = 10) of the SLF before (Baseline) and after (2, 10 and 20 min) 10 min of 2.5 mA cathodal tsDCS. The amount of facilitation from early conditioning is expressed as a percentage of the facilitation obtained during the first baseline measure. * indicates a significant difference from baseline (*p* < .05). (c) Average trace from one representative participant displaying changes in SLF without changes in test H‐reflex amplitude following tsDCS. Results are expressed as means ± *SD*

To explore at what level of the corticospinal system plastic changes were induced by cathodal tsDCS, we investigated H‐reflexes conditioned by subthreshold TMS (Figure 5a). Specifically, we investigated whether short‐latency facilitation, presumably reflecting the excitability of the fastest conducting corticospinal neurones, was changed following tsDCS. Short‐latency facilitation was significantly increased at 2 min (11.2 ± 3.5%, *p* = .004) and 10 min (12.2 ± 3.5%, *p* = .001) when compared to baseline (Figure 5b) following cathodal tsDCS. The size of the unconditioned, test H‐reflex was kept constant (*p* = .32) (Figure 5c). This indicates that tsDCS exerts some of its effects by upregulating excitability of the fastest conducting, presumably monosynaptic, corticospinal neurones.

## 4 DISCUSSION

The main finding of this study was that cathodal tsDCS in a double‐blinded, sham controlled research design facilitated ballistic voluntary contraction of ankle plantar flexors. Since Soleus MEPs and short‐latency facilitation of the Soleus H‐reflex induced by TMS were enhanced by cathodal tsDCS, whereas H‐reflexes were unchanged, we suggest that the facilitation of voluntary effort by tsDCS is due to increased corticospinal drive to spinal motor neurons.

### 4.1 Variability of effects

tDCS over the motor cortex has shown variable electrophysiological and behavioural effects and there is still no general consensus about its usefulness as a therapeutic technique for rehabilitation (Buch et al., 2017; Huang et al., 2017; Rothwell, 2016; Terranova et al., 2018). From the relatively few published studies on tsDCS in human participants a similar lack of consistency appears to be present at least in the case of electrophysiological measures such as MEPs and H‐reflexes (Albuquerque et al., 2018; Bocci, Barloscio, et al., 2015; Bocci, Caleo, et al., 2015; Bocci, Marceglia, et al., 2015). In the present study, clear increases in MEP and in ballistic plantar flexion were observed in some participants, whereas in others, only modest or no effects were observed. This is in contrast to animal studies where clear, consistent and reproducible effects are observed (Baczyk & Jankowska, 2018; Jankowska, Kaczmarek, Bolzoni, & Hammar, 2016). There may be several reasons for the larger variability in human participants. Probably the most important is that the field elicited by tsDCS is dependent on passage of current through skin, subcutaneous tissue, muscles and bones in order to influence the neuronal elements in the spinal cord. The field strength and the efficiency of tsDCS in activating the different neuronal elements in the human spinal cord therefore will strongly depend on individual differences in tissue properties and anatomical factors, as well as a range of physical factors related to the stimulation conditions (e.g., electrode size, conductive material, placement of electrodes). Studies on the distribution of the current applied by tsDCS in human participants have also indicated that the field may vary from being insufficient to reach the spinal cord at all to being sufficiently strong to influence neuronal elements in the ventral spinal cord (Bastos et al., 2016; Fernandes, Salvador, Wenger, de Carvalho, & Miranda, 2018; Kuck, Stegeman, & van Asseldonk, 2017). There is clearly a need for improved means of monitoring the distribution and strength of the applied current in individual experiments to obtain more consistent effects and in order to determine which neuronal elements in the spinal cord are responsible for the observed electrophysiological and behavioural effects. Without such information, all considerations of the site of action of tsDCS will remain speculative.

### 4.2 Intensity and duration

We observed that the effect of tsDCS was highly dependent on stimulus intensity, but was only marginally influenced by the duration of the stimulation. The intensity‐dependency is not surprising and only indicates that a sufficiently high intensity field is necessary in order to influence the neuronal elements responsible for the observed effects. This may be related to either the anatomical distribution of the current or threshold properties of the neuronal elements in the field. The lack of relation to the duration of the stimulus is more interesting. Animal studies have suggested that very brief cathodal stimulation (seconds) is sufficient to elicit a long lasting change in excitability of the axons (Jankowska et al., 2016). We did not test such short lasting stimuli, but since even the shortest stimulus that we used (3 min) showed a near similar size of effect as much longer lasting stimuli makes it possible that similar effects are involved as in animal experiments. Future studies that explore this possibility are required. In contrast to animal studies, the changes in excitability following tsDCS in humans are not constant over time, but disappear after 10–20 min. There may be several reasons for this, but the most likely is that the observations in human participants are indirect and influenced by factors that are more difficult to control than in the case of animal experiments. Humans are awake during the experiments and the neuronal state is therefore likely to influence the effect of tsDCS, whereas animals are anesthetized during experiments and therefore have comparatively little neuronal activity (Aguilar et al., 2011).

### 4.3 Evidence of increased corticospinal drive to spinal motor neurons by tsDCS

Although the increase of Soleus MEPs following tsDCS, without a concomitant increase of Soleus H‐reflexes, is suggestive of an altered excitability of neuronal elements upstream from the spinal motor neurons (i.e., corticospinal neurons or interneurons contacted by corticospinal fibers), any comparison of compound potentials such as MEPs and H‐reflexes should be made with considerable caution. Although the H‐reflex is sensitive to excitability changes of the spinal motor neurons, it is not necessarily sensitive to excitability changes in the motor neurons that are activated in the MEP, since H‐reflexes and MEPs recorded from the same muscle may not necessarily reflect activation of the same motor neurons (Morita, Baumgarten, Petersen, Christensen, & Nielsen, 1999; Nielsen, Morita, Baumgarten, Petersen, & Christensen, 1999). The H‐reflex has also been shown to be influenced by transmission in polysynaptic pathways (such as Ib excitatory and inhibitory pathways) in addition to the Ia monosynaptic pathway and changes in excitability of interneurons in these pathways may conceal possible excitability changes in the motor neurons (Burke, Gandevia, & McKeon, 1983, 1984). Finally, the H‐reflex is also influenced by changes in the efficiency of the synapses of the Ia afferents on the spinal motor neurons and changes in presynaptic control mechanisms such as presynaptic inhibition or post‐activation depression may therefore also conceal spinal motor neuronal excitability changes (Hultborn et al., 1996; Hultborn, Meunier, Morin, & Pierrot‐Deseilligny, 1987; Nielsen, 2016).

It was consequently important that we were able to demonstrate that the short‐latency facilitation of the Soleus H‐reflex elicited by TMS was also enhanced by tsDCS. The fastest conducting corticospinal fibers with monosynaptic connections to the spinal motor neurons mediate the initial 0.5–1 ms of the short‐latency facilitation of the Soleus H‐reflex (Nielsen & Petersen, 1995a, 1995b; Nielsen, Petersen, & Ballegaard, 1995; Nielsen, Petersen, Deuschl, & Ballegaard, 1993). It has been shown repeatedly that the short‐latency facilitation of the H‐reflex is not influenced by excitability changes in the spinal motor neurons or spinal interneurons, but reliably reflects changes in transmission in fast conducting corticomotoneuronal neurons (Nielsen & Petersen, 1995a, 1995b; Nielsen et al., 1993, 1995; Petersen, Pyndt, & Nielsen, 2003). The enhancement of the initial 0.5–1.0 ms of the short‐latency facilitation therefore allows us to conclude that tsDCS has caused a change in excitability of the corticomotoneuronal neurons and since they are likely to be responsible for a large part of the MEP, this strengthens in our view that tsDCS facilitates corticospinal transmission upstream from the spinal motor neurons and interneurons.

This leaves three possible sites of action: Either excitability changes are generated in the soma or axons of the corticospinal neurons of the primary motor cortex or changes in transmission occur across their terminals in the spinal cord. The latter possibility should not be fully excluded, since evidence of changes in the efficiency of corticospinal terminals in the spinal cord has been demonstrated in relation to changes in arm posture (Donges, Taylor, & Nuzzo, 2019; Nuzzo, Trajano, Barry, Gandevia, & Taylor, 2016) and plasticity‐inducing stimulation protocols (Fitzpatrick, Luu, Butler, & Taylor, 2016; Taube, Leukel, Nielsen, & Lundbye‐Jensen, 2015). However, it would require that the current from tsDCS reaches the ventral part of the spinal cord, which is doubtful and it fails to explain why only terminals from descending fibers were influenced, whereas Ia afferent terminals were unaffected judged by the lack of effect of tsDCS on the H‐reflex. It is then more likely that tsDCS increased the excitability of the corticospinal axons, which are located relatively dorsally in the lateral part of the L1‐L2 spinal segments over which the stimulation was applied. We cannot exclude this possibility with the present experiments, but we favour the possibility that tsDCS facilitated MEPs and voluntary ballistic movements via an enhancement of sensory input to the corticospinal neurones as part of a transcortical reflex pathway (Christensen, Petersen, Andersen, Sinkjaer, & Nielsen, 2000). This would be consistent with an effect of tsDCS primarily on the most dorsal part of the spinal cord where the dorsal roots and ascending sensory pathways are located. The demonstration that cathodal tsDCS efficiently increases the excitability in ascending sensory fibers in the rat lends support to the idea that increased transmission in ascending sensory pathways with relatively direct excitatory input to the corticospinal neurones may be involved (Christensen et al., 2000). Transcortical reflex pathways have indeed been shown to be prominent for ankle muscles and to play a significant role in facilitating voluntary descending drive from the motor cortex to the spinal cord (Christensen et al., 2000). Convincing evidence that this idea is correct will require further experiments.

### 4.4 Behavioural effects

To the best of our knowledge only one other study has demonstrated behavioural effects of tsDCS (Berry et al., 2017). In that study, it was shown that tsDCS may increase power production and reduce fatigue in relation to countermovement jumps (Berry et al., 2017). Although a countermovement jump is a more complex and functional task than the isolated ballistic plantarflexion task that we investigated in this study, both tasks depend on the ability of the participant to generate significant muscle force at a specific time. It seems likely that a simple increase of excitability of neuronal elements at spinal or cortical level by tsDCS is well suited to enhance the performance in such tasks, but may be less efficient in tasks that rely more on precise muscle coordination and/or visual guidance and control. The mode of action of tsDCS might thus be similar to what has been observed for intraspinal micro‐stimulation in animal preparations (Mushahwar, Gillard, Gauthier, & Prochazka, 2002; Mushahwar & Horch, 2000). It remains to be shown whether tsDCS—similar to intraspinal micro‐stimulation—may also facilitate gait ability (Tator, Minassian, & Mushahwar, 2012).

### 4.5 Clinical implications

Regardless of the exact mechanisms and site of action, our observation of an increased ability to generate voluntary plantar flexion force during a ballistic movement suggests that tsDCS may be utilized in rehabilitation following spinal cord and brain injuries to enhance excitability at the spinal or cortical level and thereby enable surviving descending fibers to recover functional control of movement. Controlled clinical trials in selected patient populations are necessary to document whether the functional effects of tsDCS in combination with its non‐invasive and harmless mode of action would make it preferable in neurorehabilitation as compared to other means of spinal cord or brain stimulation. In view of the variable behavioural and electrophysiological effects between participants and sessions, it is necessary that the existing technology is improved in order to generate predictable activation of the relevant neuronal elements in the spinal cord and produce more reproducible effects than what we and others have observed.

## 5 CONCLUSION

We have shown in healthy humans that tsDCS can result in a behaviourally significant facilitation of voluntary central drive to spinal plantar flexor motor neurons. Since soleus MEPs and the short‐latency facilitation of soleus H‐reflexes elicited by TMS over the motor cortex, but not soleus H‐reflexes themselves, were enhanced by tsDCS, it seems likely that increased excitability of corticospinal neurones is responsible for this facilitation. We speculate that cathodal tsDCS depolarizes ascending sensory afferents in the spinal dorsal columns thereby causing increased somatosensory feedback to the motor cortex. This would make tsDCS a useful technique in the rehabilitation of motor function following central nervous lesions by enhancing activity of surviving descending pathways from the motor cortex.

## CONFLICT OF INTEREST

The authors do not report any conflict of interest.

## AUTHOR CONTRIBUTIONS

TY, ERT, CS, CF, SSG, and JL performed the experiments. TY, MMB, and JLJ performed the data analysis. TY, MMB, and JBN wrote the initial draft of the manuscript. TY, JLJ, BAC, SSG, and JBN conceptualized and designed the study. All authors critically reviewed the manuscript for intellectual content, and approved the final version of the manuscript.

## Supporting information



Supplementary Material
