# Supplementary material for: Transcutaneous spinal direct current stimulation increases corticospinal transmission and enhances voluntary motor output in humans
Source: Physiol Rep. 2020 Aug 18;8(16):e14531. doi: 10.14814/phy2.14531 (PMC7435034; doi:10.14814/phy2.14531)
Supplement: Supplementary file 1 — Supplementary Material [file PHY2-8-e14531-s001.docx]

Supplementary material

Seventeen individuals participated pilot experiments which examined the dose-response relationships of tsDCS stimulation intensity. The setup was identical to the one presented in Fig. 1. Four different stimulation intensities were studied. Specifically, current was applied using the same setup as presented in the method section with the cathode mounted at the T11-T12 spine segments and the anode positioned on the lateral side of the right shoulder. The stimulation intensities varied, and was delivered at 1 mA, 1.5 mA, 2 mA or 2.5 mA for three minutes. The order of the stimulation intensities was counter-balanced between individuals. A wash-out period was introduced between different stimulations. On a separate day, fourteen individuals returned to participate in an experiment assessing the effects of tsDCS stimulation duration. Based on the results from day 1, tsDCS was delivered at 2.5 mA for either 3 min, 10 min or 20 min in a counter-balanced fashion. A washout period of at least 40 min was introduced following each stimulation protocol to avoid carry-over effects between sessions. Corticospinal excitability was examined by applying 15 single TMS pulses of 1.2x soleus RMT prior to (~ -5 and -2 min) and following (e.g. 2, 10, 20 and 30 min) tsDCS. The average value of the peak-to-peak soleus MEP amplitudes at each time point following tsDCS was expressed as a percentage of the first baseline measure (-5 min) and used for statistical analysis.

We explored whether different tsDCS stimulation intensities and durations affected these effects. For stimulation intensity, significant changes in soleus MEP amplitudes were observed following 3 min of 2.5 mA tsDCS measured 2 min (23.0 ± 7.2%, p = 0.004) and 10 min (18.3 ± 7.2%, p = 0.03) post tsDCS. Furthermore, soleus MEP amplitudes transiently increased for 2 min following 20 min of stimulation (26.1 ± 7.1%, p < 0.001), but not for any other time point (p-value range: 0.07-0.57) (Figure S1). For stimulation intensities, 2.0 mA stimulation resulted in a significant increase in MEP amplitude observed 2 min post tsDCS (16.8 ± 7.2%, p = 0.05). No changes were observed following tsDCS at 1.5 mA (p value range: 0.19-0.99) or 1.0 mA (p-value range: 0.91-1) (Figure S1). For stimulation duration, soleus MEP amplitudes were marginally larger 2 min post tsDCS, when tsDCS was applied for 3 min (16.6 ± 7.0%, p = 0.06), but not for any other time point (0.51-0.96).


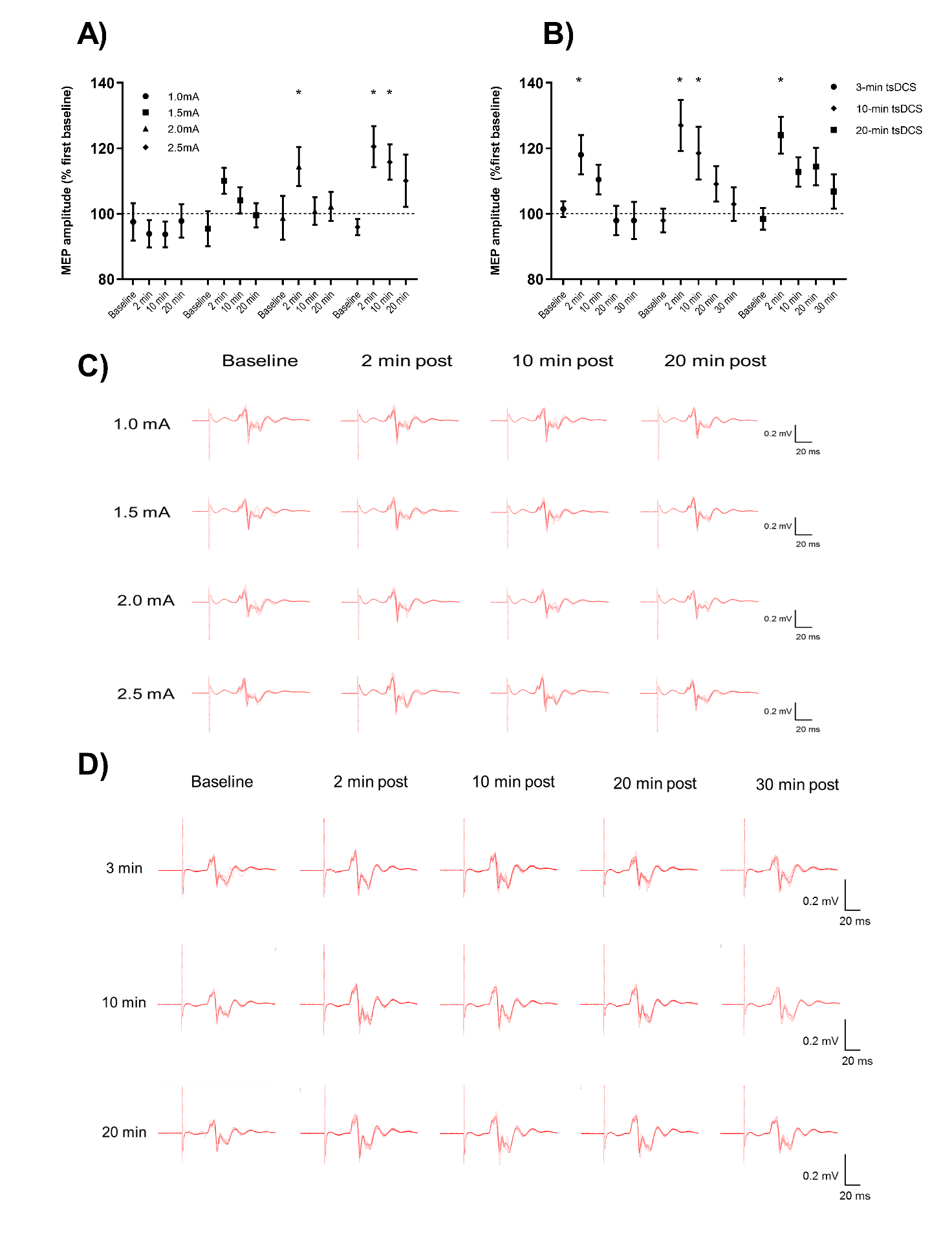


**Figure S1. Effects of cathodal tsDCS on corticospinal excitability**. **A)** Group data (n = 17) representing the normalized MEP amplitude before and after 3 min cathodal tsDCS at different stimulation intensities. **B)** Group data (n = 14) representing the normalized MEP amplitude before and after 2.5 mA cathodal tsDCS at different stimulation durations. Results are expressed as means ± sem. *indicates significant within-condition differences in MEP amplitude from baseline (p < 0.05). **C)** Soleus MEPs recorded from a single representative participant before and after cathodal tsDCS at different intensities (1.0 mA-2.5 mA). **D)** Soleus MEPs recorded from a single representative participant before and after cathodal tsDCS at different durations (3 min-20 min).
